# Influence of Sex and Cardiometabolic Risk Factors on the High-Sensitivity Cardiac Troponins at the Concentrations Used as the Thresholds for Cardiovascular Risk Stratification in a Presumably Healthy Polish Population

**DOI:** 10.3390/jcm13237126

**Published:** 2024-11-25

**Authors:** Katarzyna Bergmann, Anna Stefanska, Jacek Kubica, Magdalena Krintus, Mauro Panteghini

**Affiliations:** 1 Department of Laboratory Medicine, Nicolaus Copernicus University in Torun, Collegium Medicum in Bydgoszcz, 85-094 Bydgoszcz, Poland; zuzanna@cm.umk.pl (A.S.); krintus@cm.umk.pl (M.K.); mauro.panteghini@cm.umk.pl (M.P.); 2 Department of Cardiology and Internal Diseases, Nicolaus Copernicus University in Torun, Collegium Medicum in Bydgoszcz, 85-094 Bydgoszcz, Poland; jkubica@cm.umk.pl

**Keywords:** cardiovascular risk, cardiac troponins, cardiometabolic risk factors

## Abstract

**Background:** Low but detectable cardiac troponin (cTn) concentrations may reflect cardiovascular (CV) risk in a primary prevention setting. Using previously described thresholds for CV risk stratification, we assessed the influence of sex and cardiometabolic risk factors on the concentrations of high-sensitivity cTn in presumably healthy subjects. **Methods:** The prospective study included 597 presumably healthy individuals (313 women, 284 men). In all participants, hs-cTnI, hs-cTnT, lipid profile, C-reactive protein, glycated hemoglobin, estimated GFR (eGFR) and B-type naturetic peptide (BNP) were measured. Subjects were categorized into two groups of CV risk, based on hs-cTn non sex-specific cut-off of 5.0 ng/L. For hs-cTnI, sex-specific cut-off values were also used: ≥4.0 ng/L for females and ≥6.0 ng/L for males. **Results:** Increased CV risk, indicated by hs-cTn concentrations ≥ 5.0 ng/L, was significantly associated with age > 40 years, male sex, obesity and BNP concentrations ≥ 35 ng/L. Using the same 5.0 ng/L threshold, hs-TnT classified approximately twice as many individuals into the CV subgroup compared to hs-cTnI, particularly in males (31% vs. 13%, respectively). After applying sex-specific cut-offs for hs-cTnI, the proportion of females and males with increased risk became similar (8% vs. 9%, respectively). In contrast, using non-sex-specific cut-offs for hs-cTnI resulted in a proportion of 6% for females and 13% for males. BNP and eGFR had significant impact on CV risk stratification using sex-specific cut-offs for hs-cTnI. **Conclusions:** Our findings suggest the necessity of using sex-specific cut-offs for hs-cTn as a cardiovascular risk marker, in addition to other cardiometabolic factors, in the general population.

## 1. Introduction

Troponins are proteins that regulate the contraction of skeletal muscles and cardiomyocytes. There are three isoforms: troponin I (TnI), troponin T (TnT) and troponin C (TnC), of which TnI and TnT are referred to as isoforms characteristic for the myocardium (cardiac troponins, cTn). Detection of increased levels of cardiac troponins cTnT and cTnI in blood indicates specific damage to cardiomyocytes and does not depend on damage to skeletal muscle cells. For this reason, from 2000, when acute myocardial infarction (AMI) was redefined in the consensus of The Joint European Society of Cardiology (ESC) and American College of Cardiology (ACC) Committee, cTns have been used for the diagnosis of AMI as the preferred and sensitive biomarker of myocardial necrosis [1]. According to the 4th Universal Definition of Myocardial Infarction, an AMI is indicated by an increase in cTnI or cTnT concentration above the 99th percentile upper reference limit (URL), coexisting with clinical indicators of myocardial ischemia, e.g., chest pain and electrocardiogram changes [2].

Using a previous generation of assays for cTns, the marker concentrations in the blood of healthy people were considered to be undetectable. However, using new-generation laboratory methods with higher analytical sensitivity, troponin concentrations have been detected in almost all healthy people. The latest generations of highly sensitive assays (hs-cTn) allow the detection of small areas of myocardial necrosis weighing <100 mg [3]. A meta-analysis of 28 studies enrolling 154,052 subjects without CV disease showed that circulating cTnI and cTnT can be detectable even in 80% of healthy subjects below the 99th percentile URL [4]. This ability to measure cTn concentrations in apparently healthy individuals has been exploited in evaluating this marker for estimating cardiovascular (CV) risk in the general population. Several studies have demonstrated that the CV risk is increased even in apparently healthy subjects, both females and males, depending on circulating levels of hs-cTn, even when below the 99th percentile URL [3,5,6]. However, the establishment of definitive cut-off points for CV risk stratification by hs-cTn is still a matter of debate. Variables such as sex, age, or presence of cardiometabolic risk factors (e.g., hypertension, obesity, chronic kidney disease) should be taken into account to determine specific cut-offs for reliable risk stratification [7]. Studies showed an increased risk of CV events in the general population at hs-cTn concentration ≥ 5.0 ng/mL [8,9]. Recently, Abbott Diagnostics proposed sex-specific hs-cTnI cut-off points for CV risk evaluation in healthy asymptomatic individuals. Moderate to elevated CV risk was defined as hs-cTnI ≥4.0 ng/L in females and ≥6.0 ng/L in males, respectively [10]. For hs-cTnT, no clearly defined cut-off points have been defined for each sex; however, studies confirm its higher concentrations in apparently healthy men than women [11].

In this study, we assessed the influence of sex and cardiometabolic risk factors on the concentrations of high-sensitivity cardiac troponins in a presumably healthy Polish population. The main clinical question that our study addresses is whether there are factors that have a significant impact on hs-cTn when using previously described thresholds for cardiovascular risk stratification.

## 2. Materials and Methods

### 2.1. Study Group

This was a prospective cohort study. The study was performed by selecting the final group of presumably healthy subjects from a group of 707 individuals who had previously participated in the Troponin I Familiarization Study (FAM) at the Department of Laboratory Medicine, Nicolaus Copernicus University, Collegium Medicum in Bydgoszcz, Poland [12]. All FAM individuals were recruited from the general adult population. No participants were hospitalized during recruitment. The condition for recruitment was a declaration of good health status in a questionnaire completed prior to blood sampling. Finally, the study included 597 presumably healthy white Caucasian individuals: 313 women (52.4%) and 284 men (47.6%) according to exclusion and inclusion criteria. Exclusion criteria were as follows: history of CV disease, diabetes mellitus, severe chronic kidney disease (CKD), chronic or acute inflammation and concentrations of lipid parameters in serum above the alarming levels according to the Polish recommendation [13]. Included subjects had serum C-reactive protein (CRP) < 10 mg/L, estimated glomerular filtration rate (eGFR) > 60 mL/min/1.73 m^2^, B-type natriuretic peptide (BNP) < 100 ng/L (exclusion of acute heart failure), hs-cTnI and hs-cTnT below sex-specific 99th percentile URLs (Table S1) [14,15], glycated hemoglobin (HbA_1c_) < 48 mmol/mol, low-density lipoprotein cholesterol (LDL-C) < 5 mmol/L (<190 mg/dL), total cholesterol (TC) < 7.5 mmol/L (<290 mg/dL) and triglycerides (TG) < 4.52 mmol/L (<400 mg/dL). Age and smoking habits were investigated using a medical questionnaire. Smoking status was classified as current smoker, ex-smoker and non-smoker. Height (cm) and weight (kg) were measured using standard methods. Systolic (SBP) and diastolic (DBP) blood pressures were measured twice in accordance with standard procedures, in the sitting position after at least 10 min of rest. Hypertension was defined as SBP ≥ 140 mmHg or DBP ≥ 90 mmHg, according to the guidelines of the European Society of Hypertension [16]. All hypertensive subjects (*n* = 119; 18.8%) had exclusively mild (grade 1) hypertension defined as SBP 140–159 mmHg and/or DBP 90–99. Prediabetes was defined as HbA1c 39–47 mmol/mol (5.7–6.4%) [17]. Elevated BNP was defined as BNP 35–99 ng/L [18]. Abnormal lipid levels were defined as triglycerides 1.69–3.51 mmol/L (150–399 mg/dL), LDL-cholesterol 2.97–4.90 mmol/L (115–189 mg/dL) and HDL-cholesterol < 1.16 mmol/L (45 mg/dL) for women and <1.03 mmol/L (40 mg/dL) for men, non-HDL-C ≥3.36 mmol/L (≥130 mg/dL) [13]. Mildly decreased eGFR was defined as 60–89 mL/min/1.73 m^2^ [19]. The concentration of CRP 3.0–9.9 was considered elevated, reflecting high CV risk [20]. Obesity was defined as BMI ≥30 kg/m^2^.

The study was conducted in accordance with the Declaration of Helsinki and approved by the Ethics Committee of Nicolaus Copernicus University in Torun, Collegium Medicum in Bydgoszcz, Poland (no. KB 346/2017 from 27 April 2017). Written consent forms were obtained from all participants before inclusion in the study.

### 2.2. Laboratory Tests

Fasting venous blood samples were collected from each participant in the morning (7.00–9.00 a.m.) into a 6 mL tube without anticoagulant to obtain serum and two 2 mL EDTA tubes in order to determine HbA_1c_ in whole blood and BNP in plasma (Becton Dickinson, Franklin Lakes, NJ, USA). Concentrations of HbA_1c_ and BNP were immediately determined. Serum parameters were assayed after sample centrifugation for hs-cTnI, hs-cTnT, lipid profile, CRP and creatinine. All measurements were performed in the Department of Laboratory Medicine, Nicolaus Copernicus University, Collegium Medicum in Bydgoszcz, Poland on the ARCHITECT ci8200 platform (Abbott Laboratories, Chicago, IL, USA), except hs-cTnT (Elecsys Cobas e411 analyzer, Roche Diagnostics, Rotkreuz, Switzerland).

The analytical characteristics of the compared tests for and cut-off points for CV risk assessment are presented in Table S1. Single non-sex specific cut-off point for both methods was recognized as ≥5 ng/L [8,9,21], while sex-specific cut-offs were established only for hs-TnI as follows: ≥4 ng/L females and ≥6 ng/L males [10].

### 2.3. Statistical Methods

Statistical analysis was performed using Statistica 13.3 (StatSoft Inc., Tulsa, OK, USA) and MedCalc 23.0.9 (MedCalc Software Ltd., Ostend, Belgium) software. Continuous variables are presented as the median and interquartile ranges (IQR: 25th–75th percentiles), while categorical variables are presented as percentages and numbers. The W Shapiro–Wilk test was applied to test the normality. Results were compared using the Fisher exact test (categorical variables) and Mann–Whitney U-test (continuous variables). The inter-rater reliability test was used to evaluate the agreement between two assays (hs-cTnI vs. hs-cTnT). Concordant was defined as an agreement between two assays; the results of both assays were hs cTn ≥5 ng/L or <5 ng/L. Discordant was defined as a lack of agreement between the results of the two assays. We provided Kappa values to indicate the statistical strength of concordance/discordance. The Kappa value was interpreted as follows: <0.20 poor strength of agreement, 0.21–0.40 fair, 0.41–0.60 moderate, 0.61–0.80 good, 0.81–1.00 very good (Kappa (K) statistic. To evaluate the impact of the cardiometabolic risk factors on CV risk stratification by the concentration of hs-cTn, the univariable and multivariable logistic regression was performed. Parameters with non-Gaussian distribution were normalized by natural log transformation. The significance of the logistic models was tested by the Wald chi-squared statistic. The level of statistical significance was set as 0.05.

## 3. Results

The general characteristics of the study group are shown in Table 1. Significantly higher concentrations of hs-cTnI were found in males compared to females, as were LDL-C, non-HDL-C, TG, TG/HDL-C, BMI and eGFR values. Prevalence of cTn concentrations below the limit of detection (LoD) was similar in men and women for hs-cTnI, whereas for hs-cTnT, it was over 40% more common in women.

### 3.1. The Impact of Sex on hs-cTn Concentration at Established Cut-Off Values for CV Risk Stratification

We evaluated the occurrence of increased CV risk according to the adopted single (non-sex-adjusted) cut-off for troponins ≥ 5.0 ng/L. The proportion of individuals stratified into the same risk subgroup was significantly different between the two troponins (82.6% (493/597) for hs-cTnT and 90.6% (541/597) for hs-TnI; *p* < 0.0001. Totals of 104 and 56 individuals were classified into subgroups with higher CV risk by hs-cTnT and hs-TnI, respectively. Thus, the results showed that hs-cTnT > 5.0 ng/L classifies about twice as many people into the subgroup with higher CV risk than hs-cTnI. When stratified by sex, we observed significantly different proportions of males in risk groups between assays (*p* < 0.0001), while the proportion of females did not differ significantly (Table 2).

After using the sex-adjusted cut-off for hs-TnI in the risk stratification (moderate to elevated CV risk for hs-cTnI ≥ 4.0 ng/L in females and ≥6.0 ng/L in males), we observed the same proportion of females and males in the subgroup with lower CV risk (91% vs. 92%, respectively), as well as with increased (moderate to elevated) risk (8% vs. 9%, respectively) (Table S2). For the non-sex-adjusted cut-off for hs-cTnI, this proportion was 6% for females and 13% for males (Table 2).

### 3.2. Agreement Between hs-cTnI and hs-cTnT Assays in CV Risk Stratification

**Figure 1:**
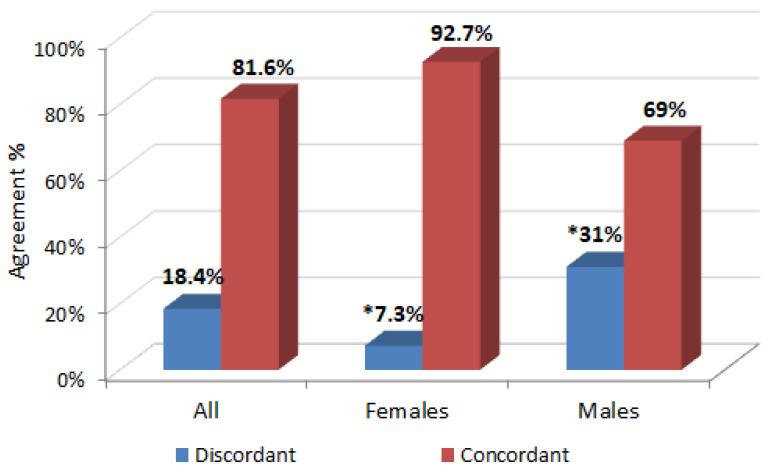
Concordance between hs-cTnI and hs-cTnT in classifying CV risk. Level of agreement: all subjects κ = 0.22 (95%CI: 0.12–0.32); females κ = 0.26 (95%CI: 0.10–0.48); males κ = 0.16 (95%CI: 0.05–0.27). * significant difference for females vs. males *p* < 0.001.

Using the single cut-off value (≥5.0 ng/L) in both assays (Abbott hs-cTnI and Roche hs-cTnT), the proportion of individuals discordantly classified into the risk subgroup was 18.4% (110/597). Totals of 4.2% (25/597) and 77.4% (462/597) subjects were concordantly classified into the subgroups with higher CV risk and lower CV risk, respectively. The weighted kappa coefficient (κ) was 0.22 (95%CI: 0.12–0.32), indicating a minimal level of agreement between assays. When the group was stratified by sex, we observed a minimal, but slightly better agreement between assays in females [7.3% (23/313) discordantly classified; κ = 0.26 (95%CI 0.10–0.48)], while the agreement became poorer in males [31.0% (87/284) discordantly classified; κ = 0.16 (95%CI: 0.05–0.27)] (Figure 1).

### 3.3. The Impact of Cardiometabolic Risk Factors on the hs-cTn Concentrations

The hs-cTn concentrations, corresponding to the thresholds for cardiovascular risk stratification (hs-cTn ≥ 5.0 ng/L), were significantly affected by age (>40 years), male sex, obesity, prediabetes, hypertension, elevated BNP concentration (35–99 ng/L), mildly decreased eGFR (60–89 mL/min/1.73 m^2^) and abnormal TG, LDL-C and non-HDL-C concentrations (significant only for hs-cTnT) (Table 3). In a multivariable model, the relation between both hs-cTn and age, obesity, male sex, elevated BNP and mildly decreased eGFR (only for hs-cTnI) remained significant. The logistic analysis also showed the impact of male sex and older age (age > 40 years) was over two-fold stronger for hs-cTnT ≥ 5.0 ng/L occurrence in comparison to hs-cTnI.

The use of sex-specific thresholds of hs-cTnI for CV risk stratification [10] attenuated the impact of confounding factors on the CV risk rating decision. Significant impact was observed only for two confounding factors: mildly decreased eGFR (eGFR 60–89 mL/min/1.73 m^2^) and elevated BNP 35–99 ng/L (Table 4).

## 4. Discussion

The influence of sex on hs-cTn concentrations is one of the most frequently debated factors determining the biological variability of this analyte [22]. In this study, we attempted to determine the impact of sex on CV risk stratification based on hs-cTnI and hs-cTnT concentrations. Our analysis showed a positive association between male sex and the classification of individuals into the subgroup with CV risk by cTns results. We also observed that the association with male sex was markedly stronger when the CV risk was stratified by hs-cTnT than by hs-cTnI. The use of sex-adjusted cut-offs for hs-cTnI eliminated the influence of male sex on risk stratification. Previous studies have pointed out the need to use separate cut-off points for women and men for diagnosing AMI [22]. The 99th URL values of hs-cTn for the methods used in this study were approximately 1.87 to 2.06 times higher in men compared to women [14,15]. Similar trends were observed for hs-cTn values for CV risk assessment in the general population. In a study enrolling 19,501 participants, Kimenai et al. observed significant differences between women and men by using both Abbott hs-cTnI (1.5 vs. 2.5 ng/L; *p* < 0.001) and Roche hs-cTnT (3.0 vs. 4.6; *p* < 0.001). The thresholds proposed by the authors for hs-cTnI and hs-cTnT (2.1 ng/L and 6.0 ng/L in women and 2.5 ng/L and 9.0 ng/L in men, respectively) were associated with a doubling of the 5-year risk of CV events [11]. In the Whitehall II study, Siemens hs-cTnI was measured over a 15-year period in 10 308 British individuals aged 35–55 years. Initial hs-cTnI concentrations were lower in women compared to men (2.4 vs. 3.7 ng/L; *p* < 0.001). The authors observed an increase in hs-cTnI values with advancing age; however, concentrations remained lower in females compared to males over the entirety of middle-to-late adulthood. Women showed a higher relative increase from 46 years of age and reached equivalent hs-cTnI levels approximately a decade after men [23]. This trend was also confirmed by the study by Mastali et al., which included 400 healthy subjects aged 18–86 years [24]. Concentrations of cTnI were measured using a very sensitive assay (Simoa HD-1, Quanterix Corporation, Lexington, MA, USA), with detectable levels of hs-cTnI found in 95% of participants. Females had lower median hs-cTnI concentrations compared with males (0.43 ng/L vs. 0.83 ng/L; *p* < 0.001). Both sexes demonstrated significantly higher hs-cTnI concentrations above 40 years of age, although in men, it remained about 1.6 times higher than in women [24]. Sex differences in hs-cTn were also described by Giannitsis et al. [25]. In this study, the 99th percentile URL was determined in a population of 827 presumably healthy individuals. Both Roche hs-cTnT and Abbott hs-cTnI showed significant sex differences, with values ranged 13.1–13.3 ng/L (females) and 16.8–19.9 ng/L (males) for hs-cTnT, and 10.3–12.5 ng/L (females) and 27.4–29.7 ng/L (males) for hs-cTnI, depending on the definition of health status.

Although the exact mechanisms associated with the sex differences in hs-cTn are not fully understood, several hypotheses have been described to explain this phenomenon. Due to the fact that troponin concentration reflects the amount of damaged myocardium, the difference in hs-cTn levels might be related to sex-specific variations in body composition, cardiac mass and rate of cardiomyocyte apoptosis. Moreover, the pathophysiological mechanism of cardiac ischemia, the response to ischemia and reperfusion and the grade of coronary atherosclerosis seems to be different in men and women. There is also evidence that estrogens, with their anti-inflammatory and antioxidant properties, have a protective role on the myocardium and reduce cardiomyocyte injury [26].

Results from our study, as well as findings from other population-based studies, support the statement that hs-cTn cut-offs for reliable CV risk assessment should be sex-specified and their establishment for hs-cTnT is required. Moreover, further analysis regarding age-adjusted thresholds for the general population seems to be necessary. Additionally, in our study, we found discordance between hs-cTnI and hs-cTnT assays in classifying subjects into CV risk subgroups, with a higher rate of discordant classification results observed in the male group. Although the issue of discordance between assay methods was analyzed in the context of determining the 99th percentile URL for the diagnosis of AMI [27,28,29,30], there are few studies examining the agreement between hs-cTnI and hs-cTnT measurements for screening CV risk in healthy individuals. A modest correlation between these analytes was found in ambulatory participants (r =  0.585; 95%CI 0.562–0.608), with a concordance of dichotomized hs-cTnI and hs-cTnT estimated by κ statistic of 0.397 [31]. McEvoy et al. compared three assays for hs-cTnI (Abbott, Siemens and Ortho) and one for hs-cTnT (Roche) for their ability to stratify CV risk in the general population [32]. The authors concluded that correlations between assays were modest.

The assessment of the relationship between hs-cTn concentrations and other markers of cardiometabolic disorders in the context of CV risk stratification is one of the most important contemporary clinical problems, taking into account the increasing trend in the incidence of obesity, diabetes and metabolic syndrome (MetS) that has been continuing for several decades. Cardiometabolic syndrome, due to the complexity of its components, has a multifaceted impact on the development of CV disorders and may be associated with a release of cTn from damaged myocardium [33,34,35].

Several studies have described the relationship between hs-cTn and cardiometabolic disorders in the general population. The prevalence of detectable hs-cTnT concentrations (>5.0 ng/L) was higher in subjects with MetS [36]. Abdominal waist, plasma glucose and hypertension were the components of MetS that were more strongly associated with elevated hs-cTnT concentrations. Sugiura et al. obtained similar results in subjects with MetS by using Abbott hs-cTnI [37]. Concentrations of hs-cTnT were also significantly increased in obese subjects compared to non-obese subjects (mean, 8.0 vs. 6.0 ng/L; *p* < 0.001) and positively associated with the occurrence of obesity even after adjustment with other CV risk factors [38]. A study conducted in the BiomarCaRE cohort (74,738 participants) revealed significantly higher Abbott hs-cTnI concentrations in individuals with diabetes than in those without [39]; hs-cTnI was also positively associated with systolic blood pressure, left ventricular mass, carotid plaque thickness and correlated negatively with eGFR [39]. Wang et al. described clinical factors affecting hs-cTnT concentrations in a Chinese population [40]. A multivariate regression model showed that hs-cTnT was positively correlated with BMI, hypertension, fasting plasma glucose, dyslipidemia and renal dysfunction, while the relationship with BNP remained non-significant [40]. In the Dallas Heart Study, changes in circulating NT-proBNP and hs-cTnT were compared in a population-based cohort, including 1877 participants. Both biomarkers were associated with age, blood pressure, diabetes, eGFR and BMI; however, in a more detailed analysis of body composition, no relation with body fat distribution was seen for hs-cTnT [41]. The PROSPER study including 4402 elderly people showed that hs-cTnT was positively correlated with age and male sex, as well as with cardiometabolic risk factors, such as BMI, blood pressure, NT-proBNP, CRP, presence of diabetes mellitus, and is inversely correlated with eGFR [42]. In the study by de Bakker et al. [23], hs-cTnI was significantly related to the occurrence of diabetes, dyslipidemia, smoking and increase in blood pressure and BMI in a 15-year observation in both sexes. Interestingly, in multivariate regression models, BMI was more strongly associated with hs-cTnI in men than women (*p* = 0.008), while diabetes was associated with increased hs-cTnI concentrations in women, but an inverse correlation was found in men. These reports indicate that the use of hs-cTn for population-based CV risk screening may require the development of cut-off points that take into account the presence of cardiometabolic risk factors.

In our study, hs-cTns were significantly related to age > 40 years, obesity, decreased eGFR and increased BNP. Moreover, we found an association between both hs-cTnI and hs-cTnT with prediabetes and increased non-HDL-C; however, after adjustment for age, sex, BMI, smoking and hypertension, these factors remained insignificant. Many studies confirmed the significant impact of age on hs-cTnI and hs-cTnT in the general population, as well as in patients with suspected AMI [24,43,44]. In the Canberra Heart Study, significantly higher hs-cTnI concentrations, especially >90th percentile URL, were more frequent in the group of apparently healthy individuals over 65 years of age, both in women and men [45]. These differences showed a relevant impact on the distribution of hs-cTnI concentration in the population, which suggests the need to adjust the cut-off points for the diagnosis of cardiovascular events in elderly subjects. A study on the development of 99th percentiles for different troponin assay methods conducted in the Danish population showed the need to modify decision values for subjects >50 years of age [46]. The influence of age on troponin concentration was particularly strong for hs-cTnT in individuals > 70 years of age. Numerous studies have also highlighted the relationship between increased hs-cTn concentration and the degree of renal function impairment [47]. The results from an EQUAL Study indicated that in patients with stable chronic kidney disease (CKD), hs-cTnT concentration was inversely correlated with eGFR value [48]. Each 15 mL/min/1.73 m^2^ lower mean eGFR was independently associated with a 23% higher baseline hs-cTnT and a 9% steeper increase in hs-cTnT. In a community-based Chinese population study, low eGFR levels were associated with detectable hs-cTnT concentrations, and this relationship was particularly significant in subjects with moderate-to-severe reduced renal function [49]. To our knowledge, there is a lack of studies on establishing hs-cTn cut-offs for CV risk stratification in the general population, particularly those taking into account eGFR. However, recently, Chen et al. proposed kidney function-specific hs-cTnT cut-off values for AMI diagnosis, determined as 14, 18 and 48 ng/L for eGFR values >60, 60–30 and <30 mL/min/1.73 m^2^, respectively [50]. In our study, we observed that the use of sex-adjusted cut-off points for hs-cTnI eliminates the influence of most confounding factors on CV risk stratification, except for lower eGFR values and higher BNP concentrations. These results lead us to conclude that using sex-adjusted cut-off points for Abbott hs-cTnI reduces the influence of age and most cardiometabolic disorders on CV risk assessment, potentially aiding clinicians in their decisions regarding further patient management.

The use of hs-cTn for primary prevention and assessing CVD risk in the general population has not been proposed so far in routine clinical practice, in both European and American recommendations. The concentration of hs-cTn is considered the novel independent risk factor for incident cardiovascular disease [51]. However, there are currently no studies that could confirm the additional benefit of using this marker in comparison to the traditional risk factors. A detailed meta-analysis published in 2024 by Neumann et al. reported that the improvement in CVD risk prediction in the general population was relatively small when hs-cTn was added to traditional risk equations [52]. Therefore, the use of hs-cTn in routine practice for CV risk assessment in apparently healthy individuals requires further investigation.

Our study has limitations. The evaluated group was relatively small, and our results should be verified in a larger cross-sectional study. However, considering the aim of the study, which was to evaluate the influence of gender and cardiometabolic factors on hs-cTn concentration, but not to assess the risk of cardiovascular disease in individuals, it seems that the finding of a cardiovascular event as an endpoint in a follow-up study would not have had a significant impact on the obtained results. It should also be mentioned that we used the hs-cTn cut-off points that were previously verified in follow-up studies by other authors. In this study, we tried to estimate which risk factors affect cardiovascular risk stratification using cardiac troponins and whether the use of hs-cTn sex-specific cut-off values reduces the influence of other factors in the assessment of this risk. Additionally, we compared hs-cTnI and hs-cTnT in this aspect. Using statistical tests, and above all, regression analysis as the primary outcome measure, we observed the significant impacts of sex and select cardiometabolic risk factors on both hs-cTnI and hs-cTnT using the single cut-off point for CV risk stratification.

On the other hand, the differences observed in hs-cTnI and hs-cTnT assays appear promising and highlight the need to adjust thresholds for hs-cTn in population screening, not only based on sex but also considering the presence of other cardiometabolic risk factors such as obesity, diabetes and decreased renal function. This strategy may enable more accurate cardiovascular risk stratification in the general population if hs-cTns are implemented for this purpose.

## Figures and Tables

**Table 1:** Sex-stratified characteristics of the study group.

Variables	All *n* = 597	Females *n* = 313	Males *n* = 284	*p* F vs. M
Age [years]	41 (33–52)	41 (34–53)	42 (32–52)	0.3826
Age > 40 years, % (*n*)	55 (328)	56 (174)	54 (154)	0.6237
BMI [kg/m^2^]	25.4 (22.5–28.6)	23.8 (21.2–27.2)	26.9 (24.5–29.7)	<0.0001
BMI > 30 kg/m^2^, % (*n*)	17 (100)	12 (39)	21 (61)	0.0029
hs-cTnI < LoD, % (*n*)	31.2 (186)	32.9 (103)	29.2 (83)	0.2917
hs-cTnI [ng/L] *	2.9 (2.4–3.7)	2.7 (2.3–3.3)	3.3 (2.5–4.6)	<0.0001
hs-cTnT < LoD, % (*n*)	82.6 (493)	95.2 (298)	68.7 (195)	<0.0001
hs-cTnT [ng/L] *	6.4 (5.7–8.3)	6.3 (5.5–8.4)	6.4 (5.8–8.2)	0.8171
HbA_1c_ [mmol/mol]	35.5 (32.2–38.8)	35.5 (32.2–37.7)	36.0 (32.0–38.8)	0.5771
eGFR [mL/min/1.73m^2^]	100 (93–110)	99 (91–108)	102 (94–113)	0.0003
CRP [mg/L]	0.54 (0.19–1.68)	0.58 (0.17–1.94)	0.50 (0.22–1/43)	0.6896
BNP [ng/L]	15.5 (10.0–24.6)	17.9 (10.7–30.1)	12.2 (10.0–20.4)	<0.0001
TC [mmol/L]	5.22 (4.55–5.97)	5.15 (4.50–5.97)	5.25 (4.58–6.00)	0.6061
LDL [mmol/L]	3.72 (2.53–3.85)	3.00 (2.40–3.72)	3.36 (2.71–3.98)	0.0002
non-HDL-C [mmol/L]	3.72 (3.00–4.45)	4.65 (2.82–4.21)	4.01 (3.31–4.71)	<0.0001
HDL-C [mmol/L]	1.45 (1.21–1.73)	1.60 (1.42–1.89)	1.24 (1.09–1.45)	<0.0001
TG [mmol/L] TG/HDL-C	1.14 (0.81–1.67) 1.81 (1.14–3.05)	0.95 (0.70–1.36) 1.32 (0.90–2.04)	1.40 (1.02–1.93) 2.57 (1.71–3.81)	<0.0001 <0.0001
Current smoker, % (n)	18.3 (109)	16.3 (51)	20.4 (58)	0.2030
Hypertension, % (n) &	18.8 (112)	9.9 (31)	28.5 (81)	<0.0001

Results presented as medians (Me) and interquartile range (IQR: 25th–75th percentiles) or percentages; * Me and IQR calculated values above or equal to LoD values (for hs-cTnI 1.9 ng/L, *n* = 412; for hs-cTnT 5.0 ng/L, *n* = 104); & individuals with hypertension had grade 1 hypertension defined as SBP 140–159 mmHg and/or DBP 90–99 mmHg according to the guidelines of the European Society of Hypertension [16]).

**Table 2:** Sex-stratified comparison between hs-cTn assays based on a single cut-off point for CV risk stratification.

Subgroup	hs-cTnT % (*n*)		hs-cTnI % (*n*)		*p* TnT vs. TnI	
	F	M	F	M	F	M
Lower CV Risk hs-cTn <5.0 ng/L	95.2 (298)	68.7 (195)	94.2 (295)	86.6 (246)	0.5832	<0.0001
Higher CV risk hs-cTn ≥ 5.0 ng/L	4.8 (15)	31.3 (89)	5.7 (18)	13.4 (38)	0.5832	<0.0001

CV risk—cardiovascular risk; F—females; M—males.

**Table 3:** The impact of the selected cardiometabolic risk factors on hs-cTn concentration at established cut-off values.

Risk Factors	cTnI < 5.0 *n* = 541 (A) % (*n*)	cTnI ≥ 5.0 *n* = 56 (B) % (*n*)	*p* A vs. B	OR (95%CI)	cTnT < 5.0 *n* = 493 (C) %(n)	cTnT ≥ 5.0 *n* = 104 (D) %(n)	*p* C vs. D	OR (95%CI)
Age ≥ 40 y	53 (288)	71(40)	0.0100	2.2 (1.2–4.0) #	50 (245)	80 (83)	<0.0001	4.0 (2.4–6.7) @
Male sex	45 (246)	68 (38)	0.0010	2.5 (1.4–4.5) $	40 (195)	86 (89)	<0.0001	9.1 (5.1–16.1) @
BMI ≥ 30 kg/m^2^	16 (85)	27 (15)	0.0371	2.0 (1.0–3.7) #	14 (68)	31 (32)	<0.0001	2.8 (1.7–4.5) @
Prediabetes	25 (135)	41 (23)	0.0098	2.1 (1.2–3.7) #	24 (119)	38 (39)	0.0033	1.9 (1.2–3.0) $
eGFR < 90 mL/min/1.73 m^2^	18 (95)	32 (18)	0.0115	2.2 (1.2–4.1) $	17 (83)	29 (30)	0.0047	2.0 (1.2–3.3) $
CRP ≥ 3.0 mg/L	13 (71)	16 (9)	0.5289	1.3 (0.6–2.7)	14 (69)	11 (11)	0.4156	0.7 (0.4–1.4)
non -HDL-C ≥ 3.36 mmol/L	63 (339)	75 (42)	0.0747	1.8 (1.0–3.4) #	62 (304)	74 (77)	0.0204	1.8 (1.1–2.9) #
TG ≥ 1.69 mmol/L	23 (124)	34 (19)	0.0667	1.7 (1.0–3.1)	21 (104)	38 (39)	0.0002	2.2 (1.4–3.5) @
HDL-C < 1.16/1.03 mmol/L (F/M)	12 (66)	20 (11)	0.0875	1.8 (0.9–3.6)	10 (50)	26 (27)	<0.0001	3.1 (1.8–5.3) @
LDL ≥ 2.97 mmol/L	57 (308)	59 (33)	0.7734	1.1 (0.6–1.9)	57 (280)	59 (61)	0.7078	1.1 (0.7–1.7)
Elevated BNP (35–99 pg/mL)	14 (68)	28 (14)	0.0056	2.4 (1.2–4.7) $	13 (60)	23 (24)	0.0089	1.9 (1.1–3.3) #
Hypertension	18 (95)	31 (17)	0.0188	2.1 (1.1–3.9) #	15 (75)	36 (37)	<0.0001	3.1 (1.9–4.9) @
Current smoker	17 (94)	27 (15)	0.0538	1.7 (0.9–3.3)	18 (90)	18 (19)	0.9113	1.0 (0.6–1.7)

F/M—females/males; OR—odds ratio for hs-cTn ≥ 5.0 ng/L; # *p* < 0.05; $ *p* < 0.01; @ *p* < 0.001.

**Table 4:** The impact of cardiometabolic risk factors on sex-specific thresholds for hs-cTnI [10].

Risk Factors	cTnI < 4.0/6.0 F/M *n* = 547 (E) % (*n*)	cTnI ≥ 4.0/6.0 F/M *n* = 50 (F) % (*n*)	*p* E vs. F	OR (95%CI)
Age ≥ 40 y	54 (295)	66 (33)	0.1025	1.7 (0.9–3.0)
Male sex	48 (261)	46 (23)	0.7864	0.9 (0.5–1.7)
BMI ≥ 30 kg/m^2^	16 (89)	22 (11)	0.2739	1.5 (0.7–2.9)
Prediabetes	26 (141)	34 (17)	0.2208	1.5 (0.8–2.7)
eGFR 60–89 mL/min/1.73 m^2^	18 (98)	30 (15)	0.0384	2.1 (1.0–3.7) #
CRP ≥ 3.0 mg/L	13 (72)	16 (8)	0.5492	1.3 (0.6–2.8)
non-HDL-C ≥3.36 mmol/L	63 (345)	72 (36)	0.2051	1.5 (0.8–2.9)
TG ≥1.69 mmol/L	23 (127)	32 (16)	0.1523	1.6 (0.8–2.9)
HDL-C < 1.16/1.03 mmol/L (F/M)	13 (72)	10 (5)	0.5426	0.7 (0.3–1.9)
LDL ≥ 2.97 mmol/L	57 (312)	58 (29)	0.8912	1.0 (0.6–1.9)
Elevated BNP (35–99 pg/mL)	14 (70)	26 (12)	0.0229	2.1 (1.0–4.3) #
Hypertension	18 (98)	28 (14)	0.0834	1.8 (0.9–3.4)
Current smoker	18 (96)	26 (13)	0.1647	1.6 (0.8–3.2)

F/M—females/males; OR—odds ratio for cTnI ≥ 4.0/6.0 ng/L; # *p* < 0.05.

## Data Availability

The data can be made available upon reasonable request—please contact the correspondence author. The data are not publicly available due to fact, that containing information that could compromise the privacy of research participants.

## Supplementary Materials

The following supporting information can be downloaded at: https://www.mdpi.com/article/10.3390/jcm13237126/s1, Table S1: Analytical characteristics of high-sensitivity cardiac troponin assays; Table S2: CV risk stratification with sex-adjusted cut-off of hs-cTnI.
