# Supplementary material for: Influence of Sex and Cardiometabolic Risk Factors on the High-Sensitivity Cardiac Troponins at the Concentrations Used as the Thresholds for Cardiovascular Risk Stratification in a Presumably Healthy Polish Population
Source: J Clin Med. 2024 Nov 25;13(23):7126. doi: 10.3390/jcm13237126 (PMC11642597; doi:10.3390/jcm13237126)
Supplement: Supplementary file 1 [file jcm-13-07126-s001.zip › jcm-3257439-supplementary.pdf]

**Supplementary materials**

Table S1. Analytical characteristics of highly sensitive cardiac troponin assays [14,15].

| Methods | LoB | LoD | LoQ | 99th percentile URL | Sex-specific 99th percentile URLs | | Single cut-off for CV risk | Sex-specific cut-off for CV risk | |
|---|---|---|---|---|---|---|---|---|---|
| | | | | | **F** | **M** | | **F** | **M** |
| hs-TnT (Roche) ng/L | 3.0 | 5.0 | 13.0 | 14 | 10 | 15 | 5.0 | - | - |
| hs-TnI (Abbott) ng/L | 1.3 | 1.9 | ≤3.2 | 26 | 16 | 34 | 5.0 | 4 | 6 |

LoB – limit of blank; LoD – limit of detection; LoQ – limit of quantitation; URL – upper reference limit; F – females; M – males

Table S2. CV risk stratification with sex-adjusted cut-off of hs-cTnI.

| CV Risk | Males ng/L | % (n) | Females ng/L | % (n) |
|---|---|---|---|---|
| Low | <6 | 91.9 (261) | <4 | 91.4 (286) |
| Moderate | 6-12 | 7.4 (21) | 4-10 | 7.7 (24) |
| Elevated | >12 | 0.7 (2) | >10 | 1.0 (3) |
